# Huang-Lian-Jie-Du decoction alleviates cognitive deficits in Alzheimer’s disease model 5xFAD mice by inhibiting Trem2/Dap12 signaling pathway

**DOI:** 10.1186/s13020-025-01098-x

**Published:** 2025-04-15

**Authors:** Rui-Kang Pang, Jia Shi, Xiang-Yu Peng, Shan Su, Jia-Yi Zheng, Kai Le, Vincent W. Keng, Shi-Jie Zhang, Xiao-Xiao Li

**Affiliations:** 1 State Key Laboratory of Traditional Chinese Medicine Syndrome, The Second Affiliated Hospital of Guangzhou University of Chinese Medicine, Guangzhou, 510405 China; 2 Department of Neurology, The Second Affiliated Hospital of Guangzhou University of Chinese Medicine, Guangzhou, 510000 China; 3 Department of Neurology, Guangdong Provincial Hospital of Chinese Medicine, Guangzhou, 510000 China; 4 Department of Rehabilitation Medicine, The First Affiliated Hospital of Nanchang University, No.17 Yongwaizheng Street, Nanchang, 330006 Jiangxi China; 5 Department of Rehabilitation Sciences, Faculty of Health and Social Sciences, Hong Kong Polytechnic University, 11 Yuk Choi Rd, Hong Kong, SAR China; 6 Department of Applied Biology and Chemical Technology, The Hong Kong Polytechnic University, Hung Hom, Kowloon, Hong Kong, China; 7 State Key Laboratory of Chemical Biology and Drug Discovery, The Hong Kong Polytechnic University, Hung Hom, Kowloon, Hong Kong, China; 8 State Key Laboratory of Chinese Medicine and Molecular Pharmacology (Incubation), The Hong Kong Polytechnic University Shenzhen Research Institute, Shenzhen, 518000 China; 9 College of Life Science, Zhuhai College of Science and Technology, Zhuhai, China; 10 Research Center for Chinese Medicine Innovation, The Hong Kong Polytechnic University, Hung Hom, Hong Kong, 999077 China; 11 Department of Food Science and Nutrition, The Hong Kong Polytechnic University, Hung Hom, Kowloon, Hong Kong, China

**Keywords:** Alzheimer’s disease, Huang-Lian-Jie-Du decoction, Microglia, Neuroinflammation, Trem2, Dap12

## Abstract

**Background:**

Alzheimer’s disease (AD) is a progressive neurodegenerative disorder predominantly affecting the elderly population. It is characterized by cognitive deficits associated with the accumulation of amyloid-beta plaques and neurofibrillary tangles. Huang-Lian-Jie-Du (HLJD) decoction, recognized as a representative formulation with heat-clearing and detoxification effects, has been demonstrated to be effective in treating AD. However, the underlying mechanisms require further investigation.

**Methods:**

5xFAD mice were administrated low and high doses of HLJD. The Morris water maze test was conducted to assess the effects of HLJD. Aβ42 and total tau protein levels were evaluated. Additionally, network pharmacology analysis was performed to identify therapeutic targets of HLJD’s active components and their relevance to AD. ELISA, qPCR, Western Blot, and immunofluorescence assays were employed to confirm the identified pathways. Finally, primary microglia isolated from 5xFAD mice were used to validate the candidate targets of HLJD.

**Results:**

HLJD improved cognitive deficits in 5xFAD mice and reduced amyloid plaque deposition and tau protein levels. Network pharmacology analysis indicated that HLJD influences the neuroinflammatory response, particularly through the Dap12 signaling pathway. This was confirmed by reduced levels of neuroinflammation markers, including TNF-α, IL-1β, IL-6, and indicators of microglial activation and polarization. The expression of Trem2 and Dap12 in the hippocampus (HIP) of 5xFAD mice, as well as in the isolated primary microglia, were downregulated following HLJD treatment.

**Conclusion:**

Our study indicates that HLJD alleviates cognitive deficits in AD by suppressing the Trem2/Dap12 signaling pathway in the HIP of 5xFAD mice, thereby inhibiting microglial neuroinflammation.

**Graphical Abstract:**

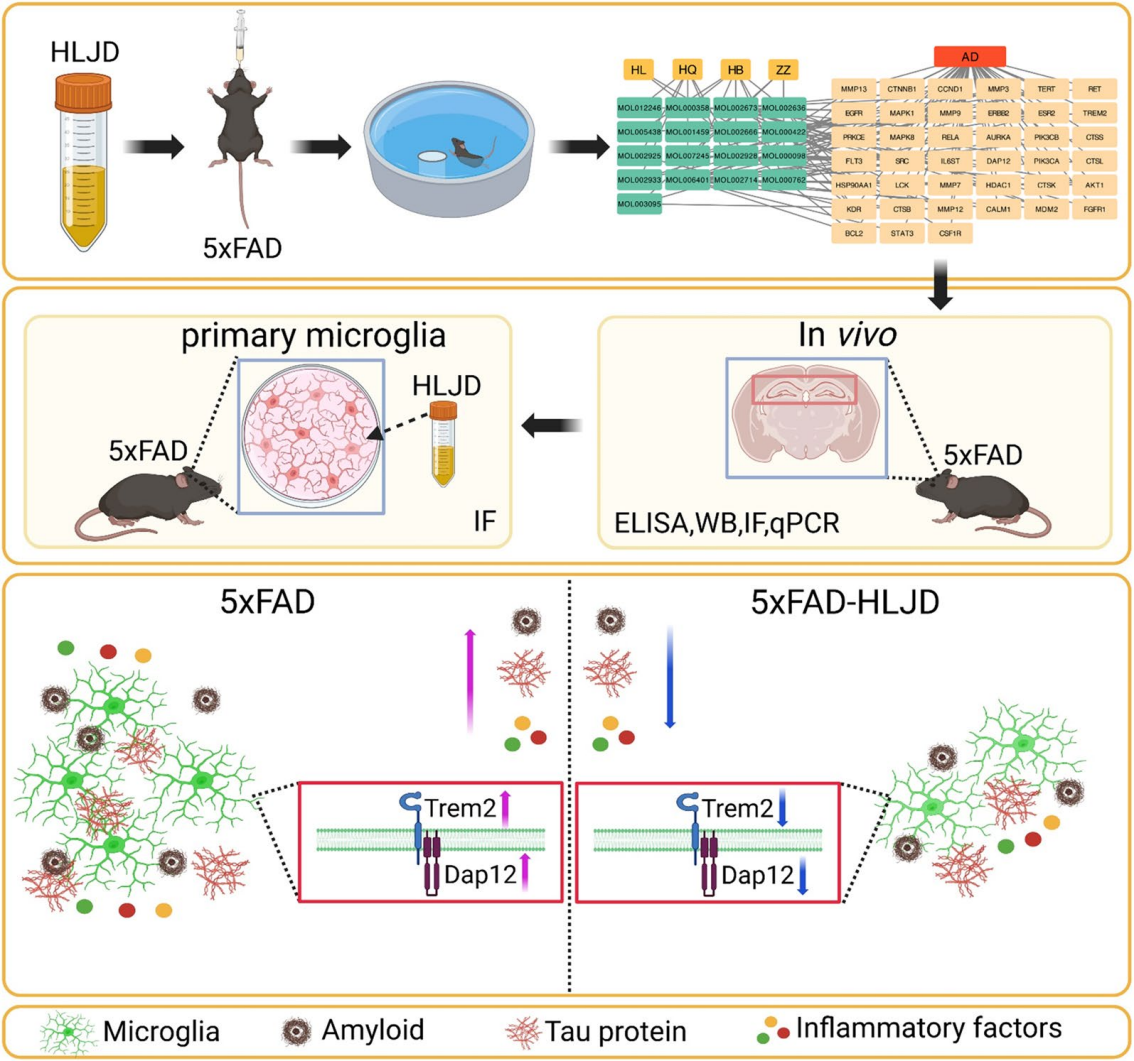

**Supplementary Information:**

The online version contains supplementary material available at 10.1186/s13020-025-01098-x.

## Article highlights

1. HLJD decoction significantly improved cognitive deficits in 5xFAD mice and reduced levels of amyloid plaque deposition and tau protein.
2. HLJD alleviated neuroinflammation in the hippocampus of 5xFAD mice by modulating the microglia activation and polarization.
3. HLJD decoction inhibiting the Trem2/Dap12 signaling pathway in 5xFAD mice and primary microglial cells.

**Supplementary Information:**

The online version contains supplementary material available at 10.1186/s13020-025-01098-x.

## Introduction

Alzheimer’s disease (AD) is an age-related neurodegenerative disorder and the leading cause of dementia in clinical practice [1]. The primary clinical manifestations of AD include impaired learning and memory functions, as well as cognitive capabilities [2]. The onset of AD is attributed to genetic factors in 60–80% of cases, with its incidence steadily increasing each year [3]. Recent projections indicate that the global incidence of AD may reach 152 million by 2050 [4]. Current therapeutic drugs for AD, such as acetylcholinesterase inhibitors and N-methyl-D-aspartate receptor antagonists, have demonstrated some efficacy in alleviating symptoms in certain clinical patients. However, no cure currently exists for AD, necessitating the exploration of alternative treatment strategies [5].

The underlying genetic mechanisms underlying AD remain elusive, with neuroinflammation recognized as a significant contributing factor [6]. Neuroinflammation may coexist with other contributing factors to AD, serving as a fundamental basis for disease progression [7]. Pathological damage within the central nervous system (CNS) induce neuroinflammation, leading to the release of inflammatory mediators such as tumor necrosis factor-alpha (TNF-α), interleukin-1β (IL-1β) and interleukin-6 (IL-6), which perpetuate the neuroinflammatory response [6]. Prolonged neuroinflammation in the CNS disrupts the balance between anti-inflammatory and pro-inflammatory processes, resulting in a sustained pro-inflammatory response that ultimately damages neurons and triggers various pathological consequences [8]. Persistent inflammation drives microglia towards a pro-inflammatory M1 phenotype while inhibiting their polarization into the anti-inflammatory M2 phenotype, further enhancing the pro-inflammatory factors [9]. In the pathogenesis of AD, the accumulation of amyloid plaques stimulates microglia activation, leading to neuroinflammation and increased secretion of TNF-α, IL-1β, and IL-6. This process impairs the ability of microglia to clear amyloid plaques, creating a vicious cycle that promotes the accumulation of amyloid plaque and neurotoxic proteins [10]. Therefore, inhibiting the neuroinflammation caused by excessive microglial activation is considered as one of the key strategies for treating AD.

Trem2 is a unidirectional transmembrane immune response receptor expressed in myeloid cells, primarily in microglia within the CNS, where it plays a critical regulatory role in microglial inflammation [11]. During microglial activation, Trem2 binds to its corresponding ligands and activates downstream regulators, including DNAX-activating protein 12 kDa (Dap12, also known as Tyrobp) [12]. Recent studies have indicated that Trem2/Dap12 receptor complex is associated with Nasu-Hakola disease (NHD), where the abnormal expression leads to the activation and proliferation of microglia in the brains of patients, impairing microglial function and exacerbating cognitive decline [13]. The Trem2/Dap12 complex recruits spleen tyrosine kinase (SYK) through a cytoplasmic immunoreceptor tyrosine-based activation motif (ITAM) [14], and SYK dysfunction has been shown to be associated with cognitive deficits in AD [15, 16]. Additionally, this complex activates glycogen synthase kinase-3 beta (GSK3β), which is considered a potential target for AD treatment [12]. The disease-associated microglia (DAM), which was initially identified in the AD mouse model of 5xFAD mice, relies on the Trem2-dependent signaling for its transformation, during which Dap12 was upregulated. Furthermore, excessive activation of DAM can negatively impact the brain if it is not controlled by negative inhibitors [17]. Therefore, targeting Trem2/Dap12 signaling may represent an important strategy for modulating microglial activation and treating AD.

According to the TCM theory, the pathogenesis of AD, particularly in its middle and late stages, is primarily associated with “fire effulgence” and “severe toxin” [18]. Professor Yongyan Wang, a renowned scholar in Chinese medicine, proposed the theory of “toxic damage to brain collaterals”, suggesting that methods involving heat-clearing, detoxification, and regulating the balance of the body's defense can nourish the brain's marrow and restore brain function, thereby treating brain diseases [19]. In this context, we infer that the "toxins" include harmful proteins, such as severely accumulated β-amyloid, which can impair the functions of brain cells, including microglia, leading to neuroinflammation during the progression of Alzheimer’s disease (AD). HLJD decoction, a traditional herbal formulation, was first documented in the “Handbook of Prescriptions for Emergencies”, also known as Zhou-Hou-Bei-Ji-Fang [20]. It consists of four herbs: *Coptis chinensis* Franch., *Scutellaria baicalensis* Georgi., *Phellodendron chinense* Schneid., and *Gardenia jasminoides* Ellis., in the proportions of 3:2:2:3. It is recognized as a key formulation for heat-clearing and detoxification, mainly used for the pattern of heat-toxin exuberance [21]. Heat-clearing addresses internal conditions related to excess heat, which may arise from external pathogens invading internal organs, while detoxification aims to weaken these pathogens and reduce their harmful effects on the body [22]. In clinical practice, the efficacy of HLJD decoction for advanced AD has been demonstrated, suggesting that detoxifying properties of HLJD may offer potential benefits for the treatment of AD [18]. In animal experimental studies, previous research has shown that HLJD decoction improved cognitive deficits in APP/PS1 mice by correcting sphingolipid metabolic disorders and reducing neuroinflammation [23]. Additionally, another study found that HLJD decoction provides neuroprotective benefits by inhibiting the NLRP3 inflammasome pathway, which helps alleviate cognitive impairments in rats with type 2 diabetes [24]. Collectively, these studies demonstrate that HLJD decoction may be effective in addressing neuroinflammation and enhancing cognitive deficits. However, the molecular mechanisms involved remain not fully understood, particularly regarding how HLJD affects microglial functions and status.

In this study, we evaluated the efficacy of HLJD decoction using 5xFAD mice. Additionally, we used network pharmacology analysis to predict the potential targets and pathways of HLJD for the treatment of AD. Through in vivo and in vitro experiments, we confirmed that HLJD decoction inhibits the Trem2/Dap12 signaling pathway and suppress microglial inflammation.

## Materials and methods

### Animals and grouping

5xFAD mice (Strain No. #034848-JAX) and C57BL/6J female mice (Strain No. N000013) were obtained from GemPharmatech (Nanjing, China). Male heterozygous 5xFAD mice were crossed with the wild type (WT) C57BL/6J female mice to produce the heterozygotes 5xFAD offspring and their WT littermates. The primers used for genotyping were as follows: common-forward primer 5’-ACCCCCATGTCAGAGTTCCT-3’, common-reverse primer 5’-ACCCCCATGTCAGAGTTCCT-3’, and mut-reverse primer 5’-ACCCCCATGTCAGAGTTCCT-3’.

All mice were housed in the Centralized Animal Facility at the Hong Kong Polytechnic University Shenzhen Research Institute, where they received humane care. They were maintained in specific-pathogen-free conditions with a 12-h light–dark cycle and had unrestricted access to standard rodent food and water. This study included four groups: a wild-type normal control group with vehicle treatment (NC-V), 5xFAD mice treated with a vehicle (5xFAD-V), and 5xFAD mice receiving low (2 g/kg) and high (4 g/kg) doses of HLJD, referred to as 5xFAD-HLJD-L and 5xFAD-HLJD-H, respectively. HLJD was given daily by oral gavage starting at three months of age and continued for seven months.

### Preparation of HLJD decoction

*Coptis chinensis* Franch. (specimen number:190601QF), *Scutellaria baicalensis* Georgi. (specimen number:200101QF), *Phellodendron chinense* Schneid. (specimen number:191001QF), and *Gardenia jasminoides* Ellis. (specimen number:190901QF) were purchased from Henan Qianfang Pharmaceutical Industry Company (China). The HLJD decoction extract was prepared according to the method described in a previous study. Briefly, the mixture of the four herbs was boiled twice with distilled water in two stages, using ratios of 1:10 (w/v) and 1:8 (w/v). The resulting filtrates were combined and concentrated using a rotary evaporator at 60 °C, followed by freeze-drying in a vacuum freeze dryer. The main components of the HLJD decoction extracts were analyzed using Ultra-Performance Liquid Chromatography Quadrupole-Time-of-Flight Mass Spectrometry (UPLC-Q-ToF-HRMS), as shown in our previous study, in which 22 components were identified [20].

### Morris water maze (MWM) test

The MWM was conducted according to the methodology outlined in a previous study [25]. On the first day, all mice were allowed to navigate the maze, which contained a visible platform to help them acclimate to the testing environment and evaluate their swimming skills. From day 2 to day 5, a colorant was added to the water to make it opaque, concealing the platform. The mice were placed in each of the four quadrants: southwest (SW), northwest (NW), northeast (NE), and southeast (SE), and allowed to explore for 60 s in each quadrant. The primary parameter observed was the escape latency, defined as the time taken by the mice to locate the platform. On day 6, the platform was removed, and the mice were placed in the water from the quadrant opposite the target platform, allowing 60 s for exploration. The time spent in the target quadrant and the number of times the mice crossed the area where the platform had been were recorded and analyzed using Noldus software.

### Collection of active components in HLJD and their potential targets

The active components of each herb in HLJD decoction were sourced from the the Traditional Chinese Medicine Systems Pharmacology Database and Analysis Platform (TCMSP) (https://old.tcmsp-e.com/tcmsp.php). A total of four herbs were analyzed, including *Coptis chinensis* Franch. (Chinese name: Huang-Lian, HL), *Scutellaria baicalensis* Georgi. (Chinese name: Huang-Qin, HQ), *Phellodendron chinense* Schneid. (Chinese name: Huang-Bo, HB), and *Gardenia jasminoides* Ellis. (Chinese name: Zhi-Zi, ZZ). The active components were selected based on the criteria of oral bioavailability (OB) ≥ 30% and drug-likeness (DL) ≥ 0.18. The SMILES notation for each compound was confirmed in the PubChem database (https://pubchem.ncbi.nlm.nih.gov). The potential targets of these active components were predicted using the SwissTargetPrediction online tool (http://swisstargetprediction.ch) based on their SMILES notations.

### Collection of AD targets and connections between HLJD and AD targets

To identify genes associated with AD, the term “Alzheimer's disease” was searched in the GeneCards database (https://www.genecards.org/), and the OMIM database (https://www.omim.org). A cutoff relevance score of > 5 was used as the selection criterion for the gene list extracted from GeneCards. The potential targets of HLJD and AD were copied into the Venny 2.1.0 (https://bioinfogp.cnb.csic.es/tools/venny/) to identify overlapping genes between HLJD and AD, resulting in the creation of a Venny diagram. The network connections among the four HLJD herbs, their active components, interesting genes, and AD were established using Cytoscape 3.10.2.

### Protein–protein interaction (PPI) enrichment analysis

The PPI enrichment analysis for those overlapping genes between HLJD and AD were conducted using Metascape (https://metascape.org/gp/index.html#/main/step1). This enrichment analysis was carried out using the following databases: STRING, BioGrid, OmniPath, InWeb_IM. However, only physical interactions in STRING (physical score > 0.132) and BioGrid are used. The Molecular Complex Detection (MCODE) algorithm has been applied to identify densely connected network components. Pathway and process enrichment analysis were applied to each MCODE component independently, and the three best-scoring terms have been retained as the functional description of the corresponding components in Supplementary Table S1.

### GO and KEGG pathway analysis

The significant targets of HLJD and AD identified through network pharmacology were further investigated to explore the potential pathways associated with both the drug and the disease. These targets were examined using the Metascape platform (https://metascape.org/gp/index.html) for the Gene Ontology (GO) and KEGG pathway analysis, with the biological species set to human. Subsequently, the resulting data files were imported to an online platform for data visualization and graphing (http://www.bioinformatics.com.cn/).

### Immunofluorescence (IF)

After euthanasia, the mice were promptly perfused with PBS, followed by a 10% formalin solution. The brains were then extracted and fixed in 10% formalin at 4 °C for 48 h. Following fixation, the brains were dehydrated in 15% and 30% sucrose solutions in PBS at 4 °C until they sank. Then, the brains were embedded in Tissue-Tek O.C.T and stored at -80 °C until further use. Coronal sections of the brain, each 30 μm thick, were prepared using a cryostat (Leica, CM 1950, Germany), and the sections were kept in PBS at 4 °C. These sections were washed three times with PBST for 5 min each, followed by blocking step using a solution containing 5% normal donkey serum and 0.35% Triton X-100 in PBS for 2 h at room temperature (RT). After blocking, sections were incubated overnight at 4 °C with primary antibodies diluted in the blocking solution. Primary antibody details as shown below: Iba1 (Abcam, ab283319, UK, 1:500), Aβ42 (Novusbio, NBP2-44113, China,1:500), Tau (Cell Signaling Technology, #46687, USA, 1:200), IL-1β (Santa Cruz, sc-52012, USA,1:500), TNF-α (Genetex, GTX110520, USA,1:500), CD169 (Abcam, ab312840, UK, 1:250), Trem2 (Thermofisher, PA5-116068, USA, 1:500) and Dap12 (Santa Cruz, sc-166084, USA, 1:500). On the next day, the sections were washed three times with PBST for 10 min each and incubated with secondary antibodies for 2 h at RT. After three washes with PBST, each lasting 10 min, the sections were mounted using a mounting medium that included DAPI. All images were obtained with a Nikon AX confocal microscope and analyzed using ImageJ software.

### Thioflavin S (ThS) Assay

The brain sections were immersed in a working solution of Thioflavin S (ThS) for 8 min at RT. Following this, the sections were dehydrated by soaking them three times in 80% and 95% ethanol, with each soak lasting 5 min. Finally, coverslips were applied with an aqueous mounting medium, and the slides were allowed to dry in the dark. ThS fluorescence signals were captured using a confocal microscope (Nikon AX) at a wavelength of 450 nm.

### Quantitative real-time PCR (qPCR)

Total RNA was extracted using a total RNA isolation kit from Promega (R6834-02, USA). The cDNA was synthesized using the Takara RT Master Mix (RR036A, Japan), followed by quantitative PCR with Forget-Me-Not™ qPCR Master Mixes (Biotium, 31042, USA) on a CFX96 Touch™ Real-Time PCR system (Bio-Rad, 1855195, USA). The expression levels of *Gapdh*, *Trem2*, *Dap12*, *Tnf-α*, *Il-1β*, *Il-6*, *Csf1*, *Csf1r*, *Il-12a*, *Nos2*, *Arg-1*, *CD206*, *Vegf*, *Il-10*, *Tgf-β1*, *Il-4*, *Il-13*, *Cxcl10*, and *Ccl3* were normalized to *Gapdh*. The sequences of the primers used are listed in Table 1.

**Table 1:**
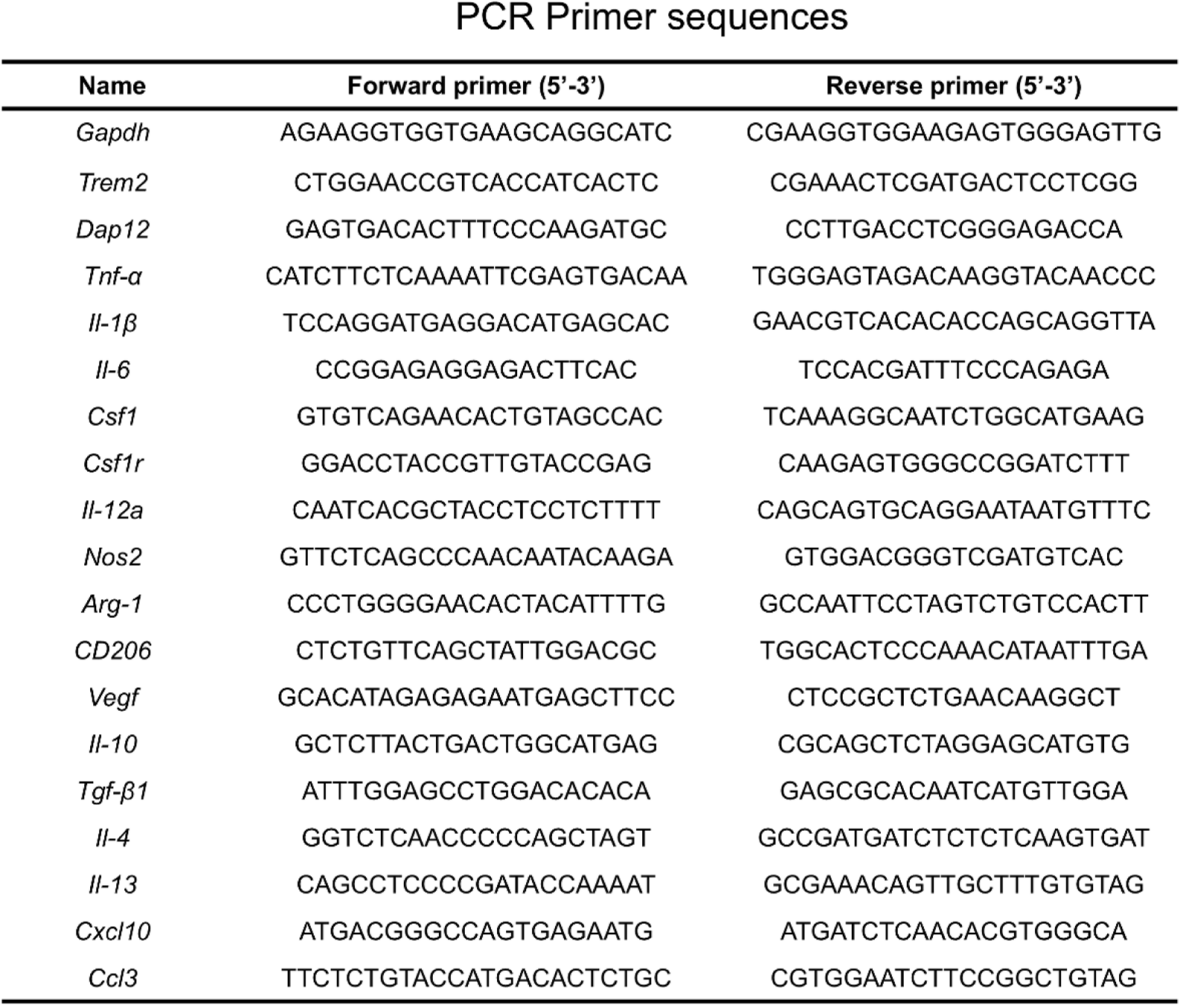
PCR primer sequences

### Western blot

The hippocampus (HIP) of the mouse brain was homogenized in RIPA buffer that included a cocktail of protease and phosphatase inhibitors. The homogenate was then centrifuged at 12,000 rpm for 15 min at 4 °C, and the supernatant was carefully harvested. Protein concentrations were measured using the BCA Protein Concentration Assay Kit (Beyotime Biological, Shanghai, China). Samples were heated at 100 °C for 10 min and separated by sodium dodecyl sulfate–polyacrylamide gel electrophoresis (SDS-PAGE). The proteins were transferred to polyvinylidene fluoride (PVDF) membranes and blocked with a 5% non-fat milk solution dissolved in TBST for one hour at RT. After blocking, the membranes were incubated with primary antibodies overnight at 4 °C, followed by washing with TBST. The membranes were then incubated with secondary antibodies in blocking buffer for one hour at RT. The specifications for the primary antibodies align with those described in the immunofluorescence protocol, while the β-Actin antibody was sourced from Santa Cruz (sc-47778). Membranes were visualized using electrochemiluminescence (ECL) (Tanon, Shanghai, China) and imaged with the Bio-Rad ChemiDoc™ imaging system (Bio-Rad, CA, USA).

### ELISA assay

The levels of TNF-α, IL-1β, IL-6 in the HIP homogenate were measured using the following kits: the mouse TNF-α ELISA kit (Solarbio, cat# SEKM-0034, China), the mouse IL-1β ELISA kit (Solarbio, cat# SEKM-0002, China), and the IL-6 ELISA kit (Solarbio, cat# SEKM-0007, China). The levels of Aβ42 in the HIP homogenate were evaluated using a mouse Aβ42 ELISA kit (Elabscience, cat# E-EL-M3010, China). All experiments were conducted following the guidelines of the assay kits.

### Primary microglia isolation and culture

The protocol for primary microglia isolation was conducted as previously described [26]. To extract primary microglial cells from mouse brains, the non-perfused brain was first chopped into small pieces and incubated in an enzymatic digestion mixture consisting of 10 mg Collagenase A (gibco, cat# 9001–12-1, USA), 70 µL DNase I (1000 U/mL, Solarbio, cat# 9001–12-1, USA), 50 µL HEPES (1 M, Servicebio, cat# G4532, China) and 1 × HBSS, bringing the total volume to 4.75 mL at 37 °C for 1 h. The enzymatic activity was neutralized by adding 250 µL of fetal bovine serum (FBS). The mixture was then centrifuged at 250 g for 5 min, and the supernatant was aspirated. The pellet was resuspended in serum-free DMEM-F12 media and gently pipetted up and down with pipette tips against the bottom of the tube until all large tissue clumps were broken down. The cell suspension was filtered through a 40 µm cell strainer, centrifuged at 250 g for 4 min, and resuspend in a 70% isotonic Percoll solution (SIP). Gradient centrifugation was performed using layers of 70%, 37%, 30% SIP and 1 × HBSS from the bottom to the top. After centrifugation at 300 g for 50 min, the interphase of 70% and 37% SIP was collected, diluted with 1xHBSS, and centrifuge at 500 g for 7 min. Finally, the pellet was resuspended in DMEM-F12 cell culture medium containing M-CSF (200 ng/mL) and seeded into a 24-well plate, where the cells were cultured for 7 days before passaging for further experiments. The primary microglia isolated from the brains of 5xFAD mice were treated with HLJD at a concentration of 50 μg/ml for 24 h to evaluate its effects on the Trem2/Dap12 signaling pathway.

### Statistical analysis

Statistical analysis was conducted using Prism 9. Data are expressed as the mean ± standard error mean (SEM), with sample sizes indicated in the figure legends. Comparisons between two groups were performed using an unpaired Student’s t-test. For comparisons involving more than two groups, one-way ANOVA was applied, followed by Tukey’s post hoc test. Significance levels were defined as follows: *P < 0.05, **P < 0.01, ***P < 0.001, ****P < 0.0001.

## Results

### HLJD decoction alleviated cognitive deficits in 5xFAD mice

**Fig. 1:**
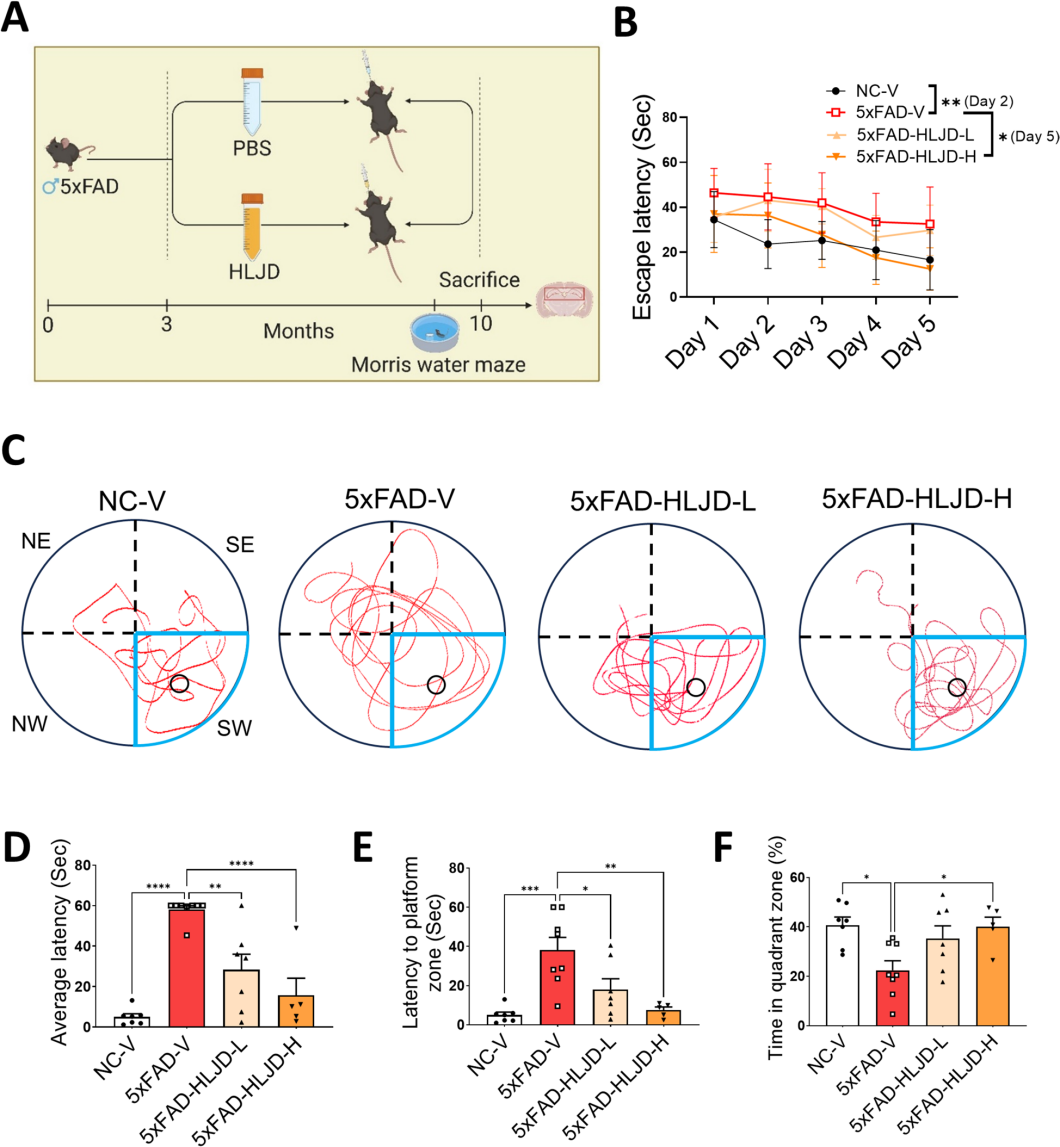
HLJD decoction alleviated cognitive impairment in 5xFAD mice. **A** Experimental timeline. **B** The escape latencies for both the visible platform on day one and the hidden platform from day 2 to day 5. **C** Representative behavioral path. **D** The average latency to target quadrant zone on day 6. **E** The latency to platform zone on day 6. **F** The time in target quadrant on day 6. Data are presented as mean ± SEM. *P < 0.05, **P < 0.01, ***P < 0.001, ****P < 0.0001

To investigate the potential benefits of HLJD decoction for the treatment of AD, different doses (2 and 4 g/kg) of HLJD decoction were administered daily by gavage to three-month-old male heterozygous 5xFAD mice and their age-matched WT controls for continuously seven months **(**Fig. 1A**)**. During the learning phase of the MWM from day 2 to day 5, the escape latency of 5xFAD-V mice was significantly longer compared to mice in NC-V group (*P* < 0.01 on day 2). High dose of HLJD decoction treatment reduced the escape latency in 5xFAD mice (*P* < 0.05 on day 5), indicating improved learning ability **(**Fig. 1B**)**. Behavioral trajectory recordings revealed that, compared to NC-V mice, the swimming paths of the 5xFAD-V mice were dispersed across all quadrants. However, HLJD intervention improved the behavioral trajectories, resulting in a greater number of records in the target quadrant **(**Fig. 1C**)**. In the testing phase on day 6, the escape latency of 5xFAD-V mice was significantly longer compared to NC-V mice (*P* < 0.0001)** (**Fig. 1D**)**, and the time to reach the platform was also markedly increased (*P* < 0.001) **(**Fig. 1E**)**. Additionally, the time spent in the target quadrant of 5xFAD-V mice was significantly reduced compared with mice in NC-V group (*P* < 0.05) **(**Fig. 1F**)**, indicating that 5xFAD mice exhibited impaired spatial memory capability. HLJD treatment significantly reversed these alterations (Average latency: 5xFAD-HLJD-L vs 5xFAD-V, *P* < 0.01, 5xFAD-HLJD-H vs 5xFAD- V, *P* < 0.0001; Latency to platform zone: 5xFAD-HLJD-L vs 5xFAD-V, *P* < 0.05, 5xFAD-HLJD-H vs 5xFAD- V, *P* < 0.01; Time in quadrant zone: 5xFAD-HLJD-H vs 5xFAD-V, *P* < 0.05) **(**Fig. 1D–F). The MWM results demonstrated that HLJD decoction effectively improved learning and memory abilities in 5xFAD mice.

### HLJD decoction reduced amyloid plaque deposition and total tau expression

**Fig. 2:**
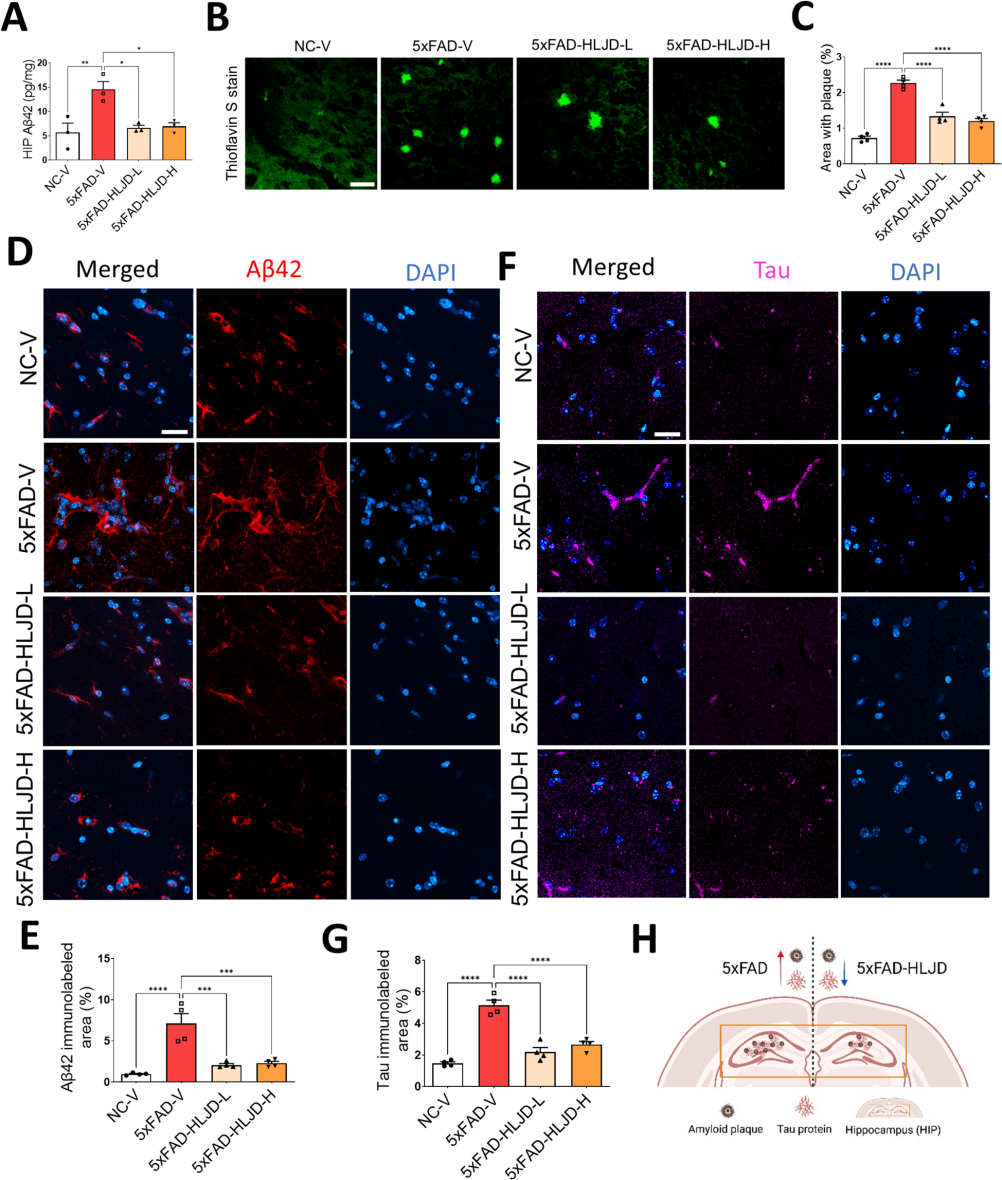
HLJD decoction reduced the expression of Aβ42 and total tau in the HIP of 5xFAD mice. **A** ELISA analyses for Aβ42 levels in the HIP of mice (n = 3). **B** Representative images of ThS staining. Scale bars, 25 µm. **C** Quantification of B (n = 4). **D** Representative images of Aβ42 staining in the HIP. Scale bars, 25 µm. **E** Quantification of Aβ42 positive area (n = 4). **F** Representative images of tau in the HIP. Scale bars, 25 µm. **G** Quantification of tau positive area (n = 4). **H** Diagram of the Aβ plaques and total tau protein levels in the HIP of 5xFAD mice treated with or without HLJD decoction. Data are presented as mean ± SEM. *P < 0.05, **P < 0.01, ***P < 0.001, ****P < 0.0001

Amyloid plaque deposition and hyperphosphorylation of tau protein are key pathologies associated with AD [27]. To investigate whether HLJD decoction can affect accumulation of Aβ42 in 5xFAD mice, ELISA assay was conducted. Our results revealed that level of Aβ42 was significantly elevated in the 5xFAD-V mice compared to that in the NC-V mice (*P* < 0.01), while HLJD treatment reduced the Aβ42 levels (*P* < 0.05 for both 5xFAD-HLJD-L and 5xFAD-HLJD-H) (Fig. 2A). ThS staining showed significantly greater deposition of Aβ plaques in the HIP of 5xFAD-V mice compared to that in NC-V mice (*P* < 0.0001), while HLJD intervention remarkably reduced the deposition of Aβ plaques (*P* < 0.0001 for both 5xFAD-HLJD-L and 5xFAD-HLJD-H) (Fig. 2B, C). Additionally, IF staining demonstrated that protein levels of Aβ42 were significantly elevated in 5xFAD-V mice compared with NC-V mice (*P* < 0.0001), and HLJD treatment decreased levels of Aβ42 (*P* < 0.001 for both 5xFAD-HLJD-L and 5xFAD-HLJD-H) (Fig. 2D, E). Furthermore, IF assay also showed higher level of total tau in the 5xFAD-V mice compared to that in NC-V mice (*P* < 0.0001), and HLJD decoction at both dosages reversed this trend (*P* < 0.0001 for both 5xFAD-HLJD-L and 5xFAD-HLJD-H) (Fig. 2F, G). Taken together, our data suggested that HLJD decoction reduced amyloid plaque deposition and total tau expression in the HIP of 5xFAD mice (Fig. 2H).

### Network pharmacology analysis of HLJD decoction in treating AD

**Fig. 3:**
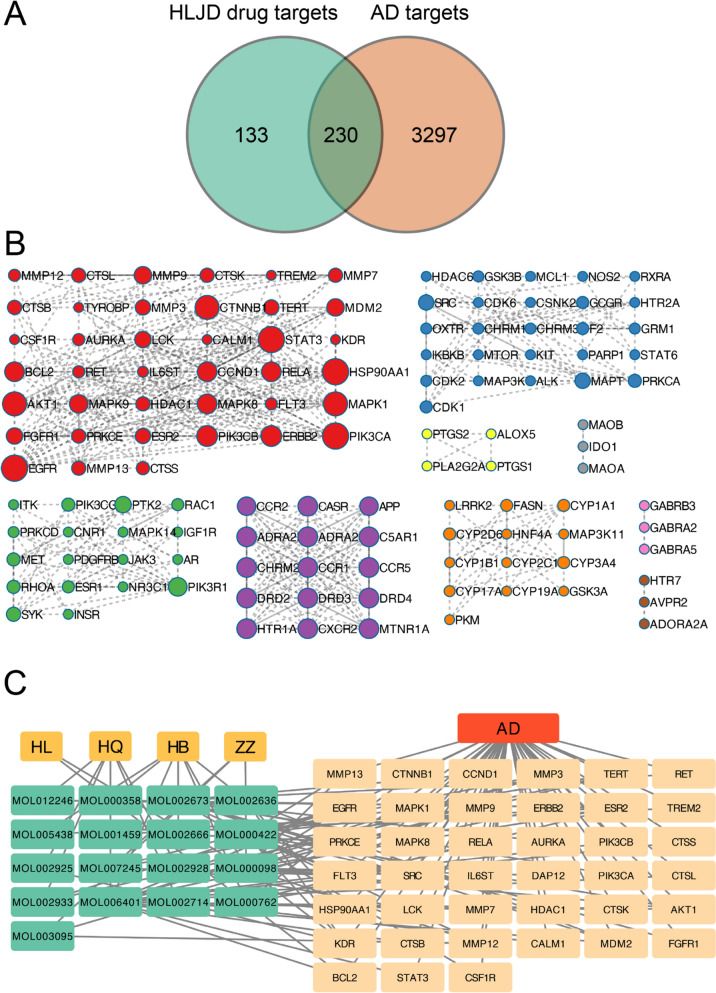
Network pharmacology diagram of HLJD for the treatment of AD. **A** Venn diagram showing targets of HLJD for AD treatment. **B** Protein–protein interaction enrichment analysis for the overlapping 230 genes using Metascape. **C** Network diagram of Traditional Chinese Medicine (yellow color)—active ingredients (green color)—intersecting genes (light yellow color) – disease (red color)

Active components from the four herbs in HLJD decoction were identified using the TCMSP online platform. Among 22 identified chemicals in HLJD decoction, 13 components were confirmed existed in at least one herb (Supplementary Table S2). Their potential targets of these active components identified in TCMSP were predicted using the SwissTargetPrediction tool. A total of 363 potential targets from the four herbs were identified, with a probability score greater than 0.12. AD targets were collected from the GeneCards and OMIM databases, resulting in a total target of 3,527 genes. The overlapping genes were calculated and illustrated using Venny 2.1.0 **(**Fig. 3A**)**. A total of 230 overlapping target genes associated with HLJD and AD were further analyzed using the Metascape online tool to identify potential pathway enriched by these genes. As shown in Fig. 3B, PPI analysis in Metascape revealed eight gene clusters, with detailed best-scoring pathway and process terms listed in Table S1. The first subcluster consists of genes considered to be interesting genes engaged by both HLJD and AD. The network of four herbs, active components, interesting targets and AD were visualized **(**Fig. 3C**)**. Within this network, baicalein, which was also identified in HLJD decoction by UPLC-Q-T oF-HRMS (MOL002714 in TCMSP), may be responsible for the effects of HLJD decoction in treatment of AD.

**Fig. 4:**
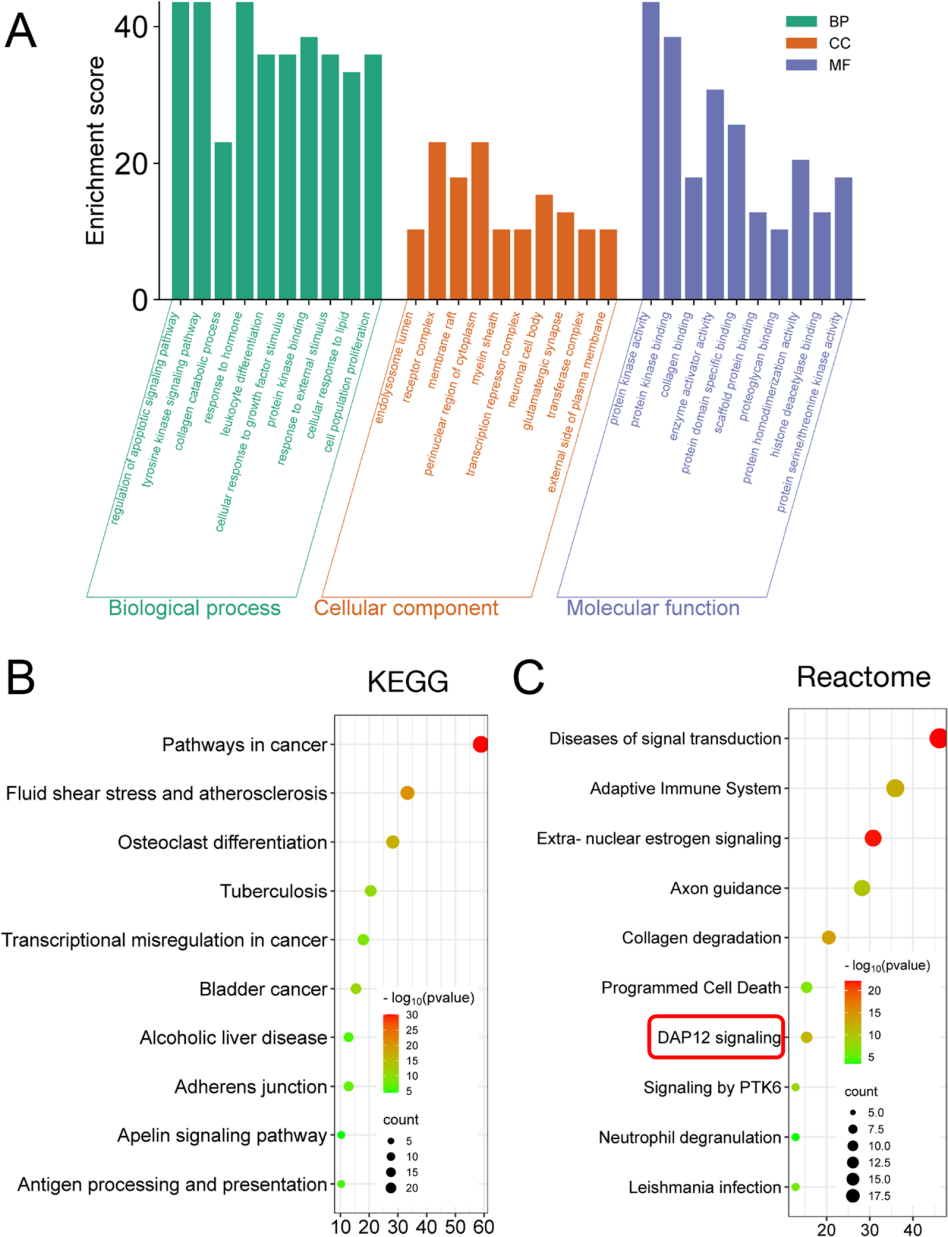
Functional enrichment analysis of the interesting target genes. Interesting target genes enriched in the top one cluster from PPI were further analyzed. **A** GO functional enrichment analysis of the interesting target genes of HLJD in the treatment of AD. **B** KEGG pathway enrichment analysis of the interesting target genes. **C** Reactome pathway enrichment analysis of the interesting target genes

To further narrow down potential pathways associated with HLJD and AD, these interesting genes were analyzed using GO and KEGG enrichment assays. The GO biological process enrichment analysis revealed that apoptosis and tyrosine kinase signaling pathways are involved **(**Fig. 4A**)**. The KEGG analysis identified pathways related to osteoclast differentiation and antigen processing and presentation, both of which are associated with inflammation **(**Fig. 4B**).** Additionally, pathways related to the adaptive immune system, DAP12 signaling, and neutrophil degranulation were identified through Reactome enrichment analysis, all of which are linked to inflammatory signaling **(**Fig. 4C**)**. Consequently, we infer that HLJD may influence the inflammatory response in 5xFAD mice.

### HLJD decoction ameliorated the neuroinflammation in the HIP of 5xFAD mice

**Fig. 5:**
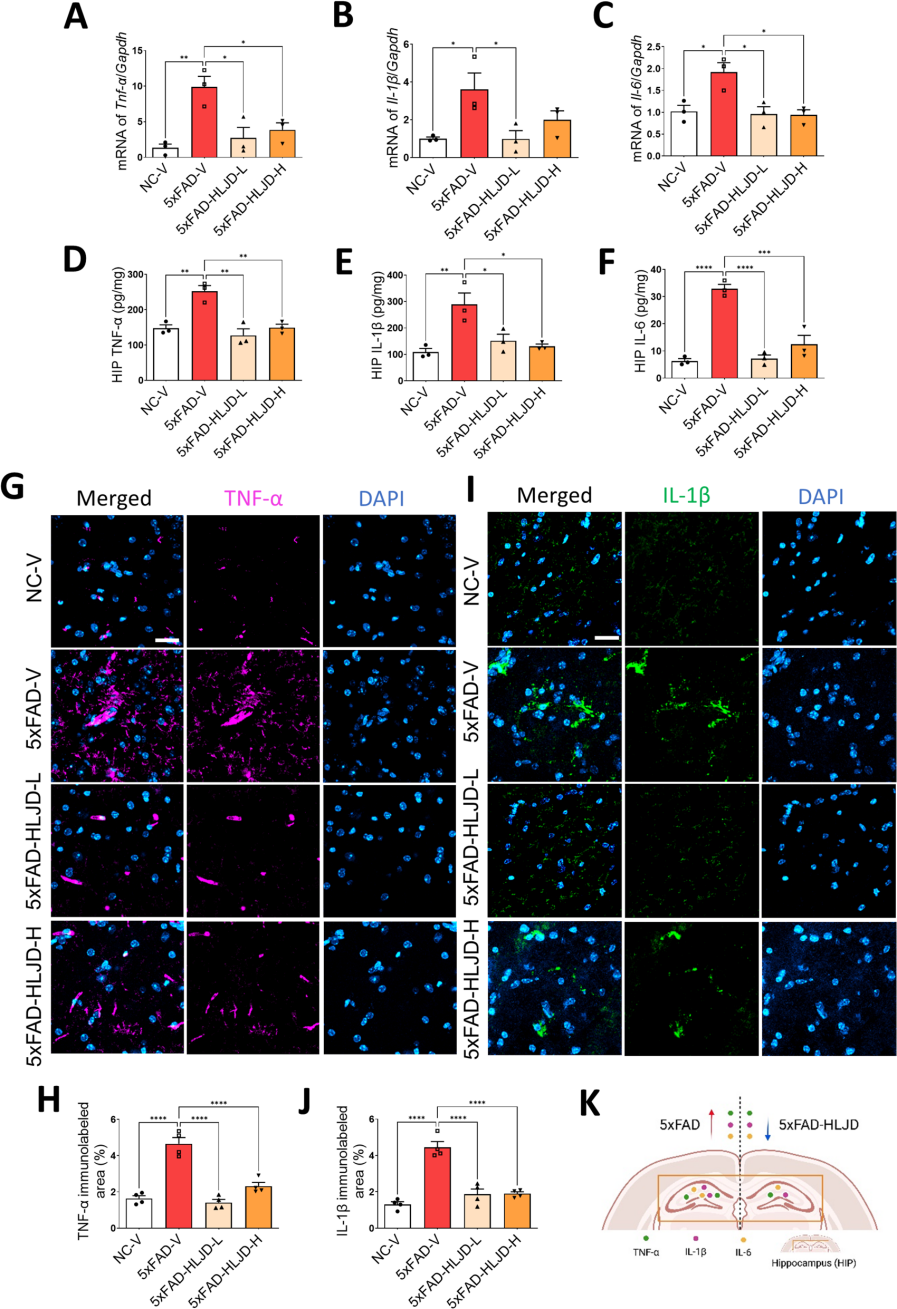
HLJD decoction ameliorated neuroinflammation in the HIP of 5xFAD mice. **A**–**C** The mRNA expressions of *Tnf-α*, *Il-1β* and *Il-6* (n = 3). **D**–**F** ELISA assays of TNF-α, IL-1β and IL-6 (n = 3). **G** Representative images of TNF-α. Scale bars, 25 µm. **H** Quantification of TNF-α immunolabeled area (n = 4). **I** Representative images of IL-1β. Scale bars, 25 µm. **J** Quantification of IL1β immunolabeled area (n = 4). **K** Diagram of the TNF-α, IL-1β and IL-6 levels in the HIP of 5xFAD mice treated with or without HLJD decoction. Data are presented as mean ± SEM. *P < 0.05, **P < 0.01, ***P < 0.001, ****P < 0.0001

To investigate whether HLJD decoction affects neuroinflammation in the HIP of 5xFAD mice, we employed qPCR assay to detect mRNA levels of inflammatory cytokines. Our results demonstrated that, the levels of *Tnf-α*, *Il-1β*, and *Il-6* were significantly increased in the HIP of 5xFAD-V mice compared to that in the NC-V mice (*P* < 0.01 for *Tnf-α*; *P* < 0.05 for both *Il-1β* and *Il-6*), and these alterations were reversed by HLJD intervention (*P* < 0.05) (Fig. 5A–C). ELISA assays showed that, compared to the NC-V mice, the levels of TNF-α, IL-1β, and IL-6 in the HIP of the 5xFAD-V mice were significantly elevated (*P* < 0.01 for TNF-α and IL-1β; *P* < 0.0001 for IL-6), and HLJD administration at both dosages reduced them significantly (*P* < 0.01 for TNF-α; *P* < 0.05 for IL-1β; *P* < 0.0001 for 5xFAD-HLJD-L and *P* < 0.001 for 5xFAD-HLJD-H in IL-6 result) (Fig. 5D–F). Similar results were obtained in the IF assay (Fig. 5G–J). Taken together, our data suggested that HLJD decoction inhibited the neuroinflammation in the HIP of 5xFAD mice (Fig. 5K).

### HLJD decoction inhibited microglial activation in the HIP of 5xFAD mice

**Fig. 6:**
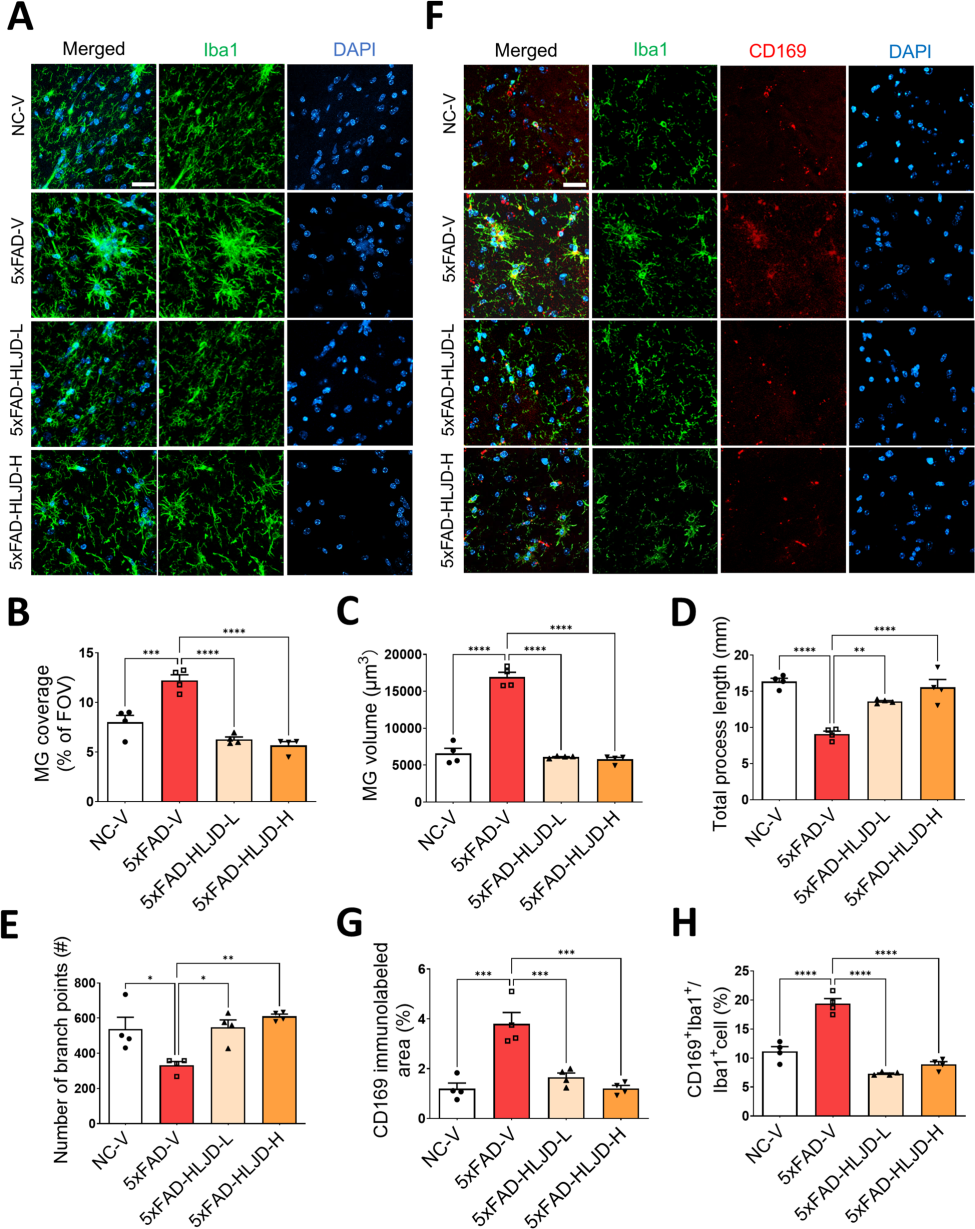
HLJD decoction alleviated the microglia activation. **A** Representative images of Iba1 in the HIP. Scale bars, 25 µm. **B** Microglia coverage (MG) for the Iba1 labelled images within the entire field of view (FOV). **C** MG volume within the FOV. **D** Total process length within the FOV. **E** Number of branch points of Iba1-labeled microglia within the FOV. **F** Representative images of Iba1 and CD169 staining in the HIP. Scale bars, 25 µm. **G** Quantification of CD169 immunolabeled area in the HIP. **H** The percentage of the CD169^+^ Iba1^+^ cells in Iba1^+^ cells (n = 4). Data are presented as mean ± SEM. *P < 0.05, **P < 0.01, ***P < 0.001, ****P < 0.0001

Microglia-mediated neuroinflammation plays a key role in the pathogenesis of AD [6, 28, 29]. To exam whether HLJD decoction affects microglia morphology in the HIP, Iba1 staining was performed. Consistent with previous findings, microglia aggregation in the HIP of 5xFAD-V mice was observed, with a higher microglial coverage (MG) percentage compared to that in the NC-V mice (*P* < 0.001), while HLJD treatment significantly inhibited these morphological alterations (*P* < 0.0001 for both 5xFAD-HLJD-L and 5xFAD-HLJD-H) **(**Fig. 6**)**. Compared to the NC-V group, the HIP microglia of 5xFAD-V mice exhibited significant increases in the microglial cell volume (*P* < 0.0001), decreased total process length (*P* < 0.0001), and reduced number of branch points (*P* < 0.05) (Fig. 6C–E). However, HLJD effectively reduced the MG cell volume (*P* < 0.0001 for both 5xFAD-HLJD-L and 5xFAD-HLJD-H), increased the total branch length (*P* < 0.01 for 5xFAD-HLJD-L and *P* < 0.0001 for 5xFAD-HLJD-H), increased the number of branches (*P* < 0.05 for 5xFAD-HLJD-L and *P* < 0.01 for 5xFAD-HLJD-H) (Fig. 6C–E). CD169, a marker of active neuroinflammation in the brain [30], indicates early microglia activation in inflammatory disorders [31]. The positive expression of CD169, along with co-localization of Iba1 with CD169, increased in the HIP of 5xFAD-V mice (*P* < 0.001 for CD169 immunolabelled area, *P* < 0.0001 for co-localization of Iba1 with CD169), but decreased following HLJD treatment (*P* < 0.001 for CD169 immunolabelled area, *P* < 0.0001 for co-localization of Iba1 with CD169). This suggests that HLJD decoction can inhibit microglia activation in the HIP of the 5xFAD mice (Fig. 6F–H).

**Fig. 7:**
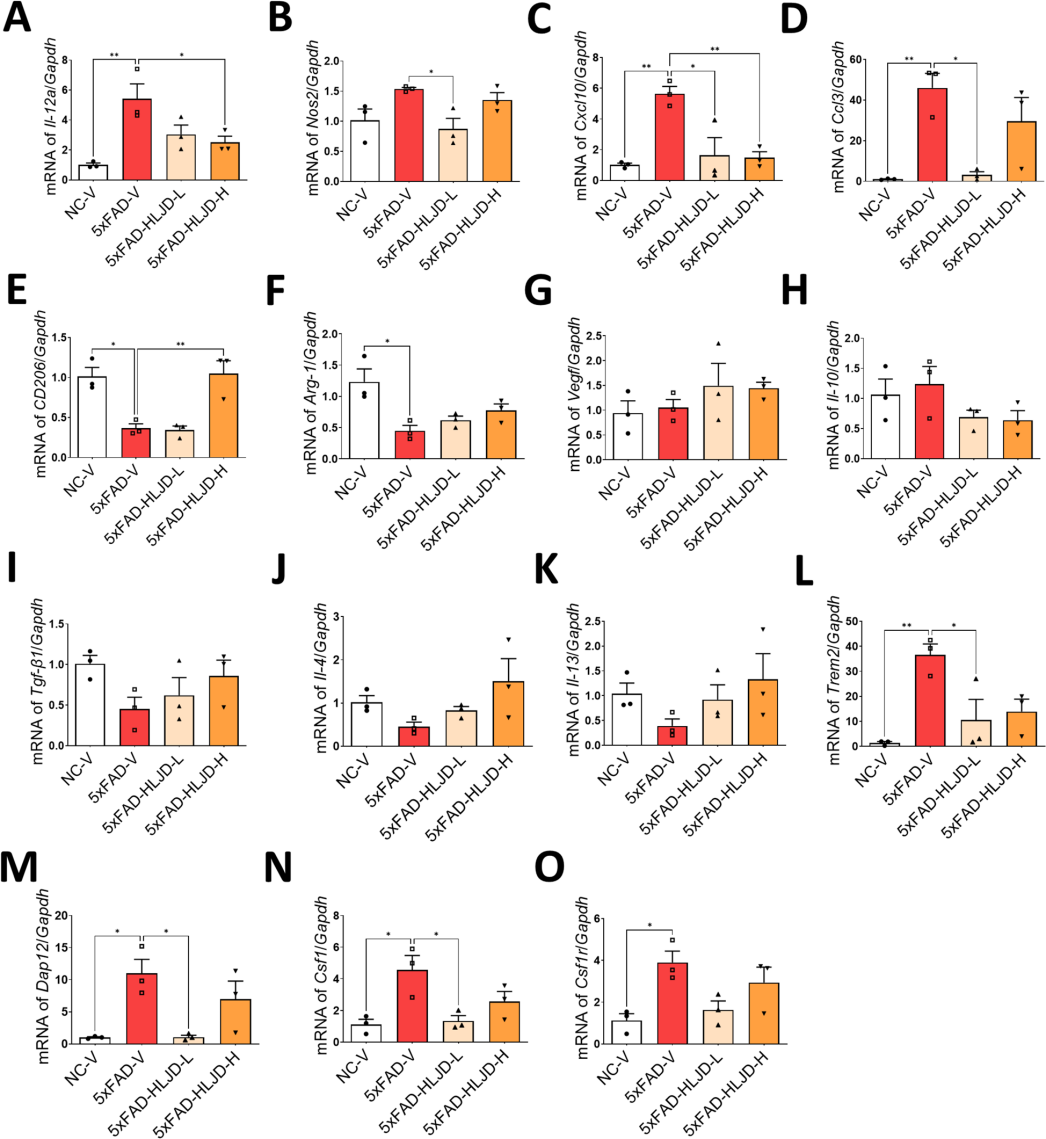
HLJD decoction affected microglial polarization and the Trem2/Dap12 signaling pathway. **A**–**D** The mRNA levels of *Il-12a*, *Nos2*, *Cxcl10*, and *Ccl3* in the HIP. **E**–**K** The mRNA expressions of *CD206*, *Arg-1*, *Vegf*, *Il-10*, *Tgf-β1*, *Il-4* and *Il-13* in the HIP. **L**–**O** The mRNA expressions of *Trem2*, *Dap12*, *Csf1* and *Csf1r* in the HIP (n = 3). Data are presented as mean ± SEM. *P < 0.05, **P < 0.01

To further explore the mechanisms by which HLJD affects microglial activation, qPCR was conducted to assess microglial polarization markers and pathways identified through network pharmacology. Our results showed that the pro-inflammatory M1 markers, including *Il-12a*, *Cxcl10* and *Ccl3*, were significantly elevated in the HIP of the 5xFAD-V mice compared to the NC-V mice (*P* < 0.01). *Nos2* also showed an increased trend, although no significant statistical differences were observed (*P* > 0.05) (Fig. 7A–D). The low dose of HLJD significantly inhibited the mRNA levels of *Nos2*, *Cxcl10*, and *Ccl3* (*P* < 0.05), while the high dose of HLJD significantly suppressed the mRNA levels of *Il-12a* and *Cxcl10* (*P* < 0.05 for *Il-12a*, *P* < 0.01 for *Cxcl10*) (Fig. 7A–D). In addition, the anti-inflammatory M2 markers, including *CD206* and *Arg-1* were significantly decreased in the HIP of the 5xFAD-V mice compared to the NC-V mice (*P* < 0.05) (Fig. 7E, F). However, there were no significant differences in the levels of M2 markers including *Vegf*, *Il-10*, *Tgf-β1*, *Il-4*, and *Il-13* among groups (*P* > 0.05) (Fig. 7G–K). These data indicates that HLJD decoction can inhibit microglial polarization towards the pro-inflammatory M1 subtype but may not effectively promote polarization towards the anti-inflammatory M2 subtype.

### HLJD decoction inhibited the Trem2/Dap12 singling pathway in the HIP of 5xFAD mice

**Fig. 8:**
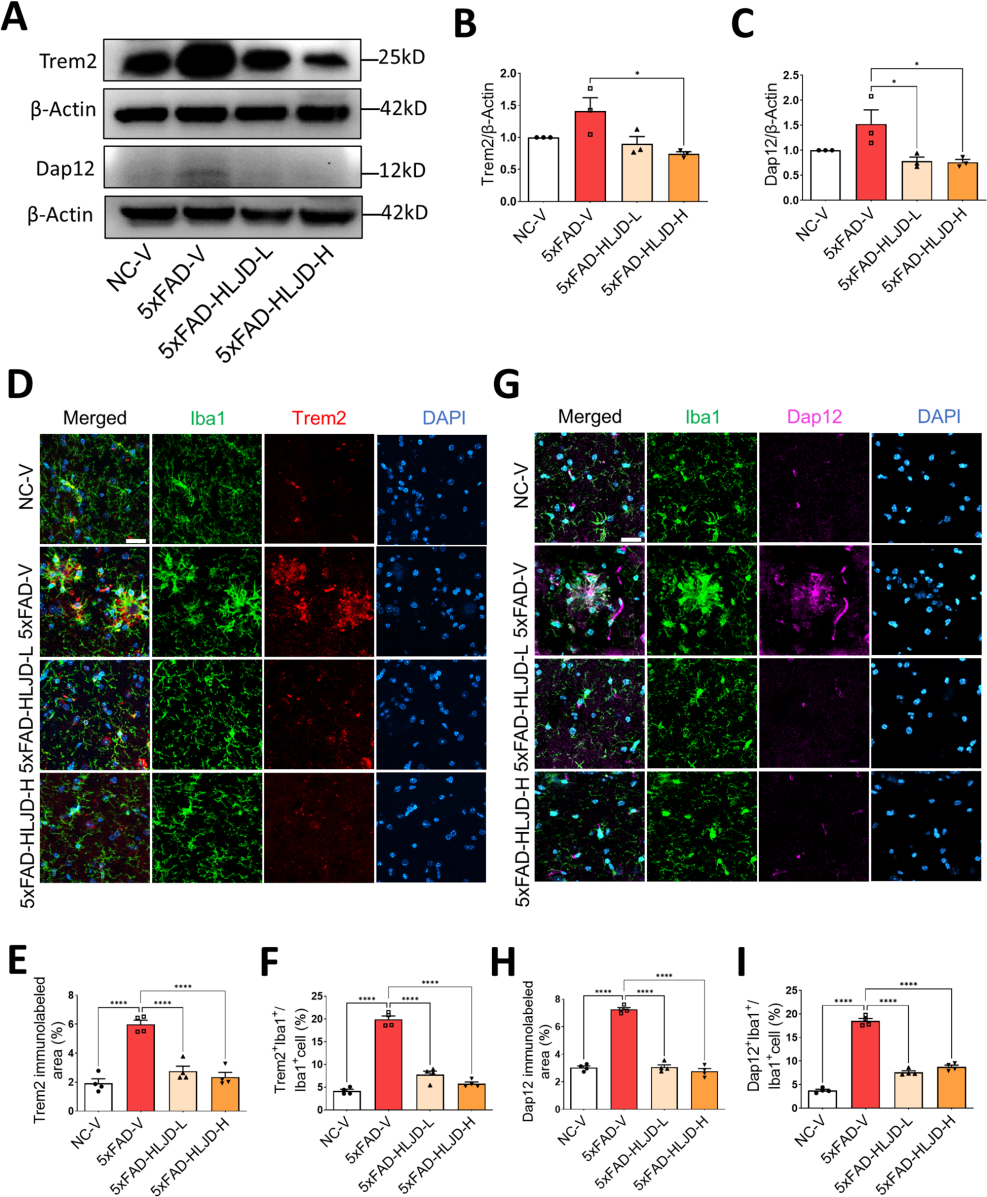
HLJD decoction inhibited the Trem2/Dap12 signaling pathway in the HIP of 5xFAD mice. **A** Representative blots of Trem2, Dap12 and β-Actin. **B**, **C** Quantification of Trem2 and Dap12 (n = 3). **D** Representative images of Iba1 and Trem2 staining in the HIP. Scale bars, 25 µm. **E** Quantification of Trem2 immunolabeled area (n = 4). **F** Percentage of Trem2^+^Iba1^+^ cells in Iba1^+^ cells (n = 4). **G** Representative images of Iba1 and Dap12 staining in the HIP. Scale bars, 25 µm. **H** Quantification of Dap12 immunolabeled area (n = 4). **I** Percentage of Dap12^+^Iba1^+^ cells in Iba1^+^ cells (n = 4). Data are presented as mean ± SEM. *P < 0.05, ****P < 0.0001

Trem2 plays a crucial role in microglial activation and the inflammatory response during the pathogenesis of AD [32]. Network pharmacology analysis suggested that the Dap12 signaling pathway may be involved in the therapeutic effects of HLJD decoction in treating AD. The Trem2/Dap12 signaling pathway, along with the Csf1/Csf1r signaling pathway, mediate microglial survival, proliferation, and the transition of monocyte to microglia [33–35]. To evaluate the effects of HLJD on these pathways, we examined the mRNA levels of *Trem2*, *Dap12*, *Csf1* and *Csf1r* in the HIP. The qPCR results indicated that *Trem2*, *Dap12*, *Csf1* and *Csf1r* were significantly elevated compared to the NC-V mice (*P* < 0.01 for *Trem2*, *P* < 0.05 for *Dap12*, *Csf1* and *Csf1r*), while HLJD-L significantly inhibited *Trem2*, *Dap12* and *Csf1* (*P* < 0.05). However, it did not significantly affect Csf1r, although a decreasing trend was observed (*P* > 0.05) (Fig. 7L–O). We further confirmed that the protein levels of Trem2 and Dap12 in the HIP of 5xFAD-V mice exhibited an upward trend compared to the NC-V group, but no statistical differences were observed (*P* > 0.05). HLJD at low dose showed a trend to reduce the levels of Trem2, and it significantly suppressed the expression of Dap12 protein levels (*P* < 0.05). HLJD at high dose significantly inhibited the expression of both Trem2 and Dap12 protein levels (*P* < 0.05) (Fig. 8A –C). The positive signal of Trem2 and the percentage of Trem2^+^ microglia in the HIP was significantly elevated in 5xFAD-V mice compared to the NC-V group (*P* < 0.0001), with this trend reversed following HLJD treatment (*P* < 0.0001 for both 5xFAD-HLJD-L and 5xFAD-HLJD-H) (Fig. 8D–F). Consistently, the positive signaling of Dap12 and the percentage of Dap12^+^ microglia elevated significantly in 5xFAD-V mice compared to the NC-V group (*P* < 0.0001), and HLJD treatment reversed this trend (*P* < 0.0001 for both 5xFAD-HLJD-L and 5xFAD-HLJD-H) (Fig. 8AG–I). Collectively, these data suggests that HLJD decoction inhibited microglia inflammation, potentially through the modulation of Trem2/Dap12 signaling in the HIP of 5xFAD mice.

### HLJD decoction inhibited the Trem2/Dap12 singling pathway in the primary microglia of 5xFAD mice

**Fig. 9:**
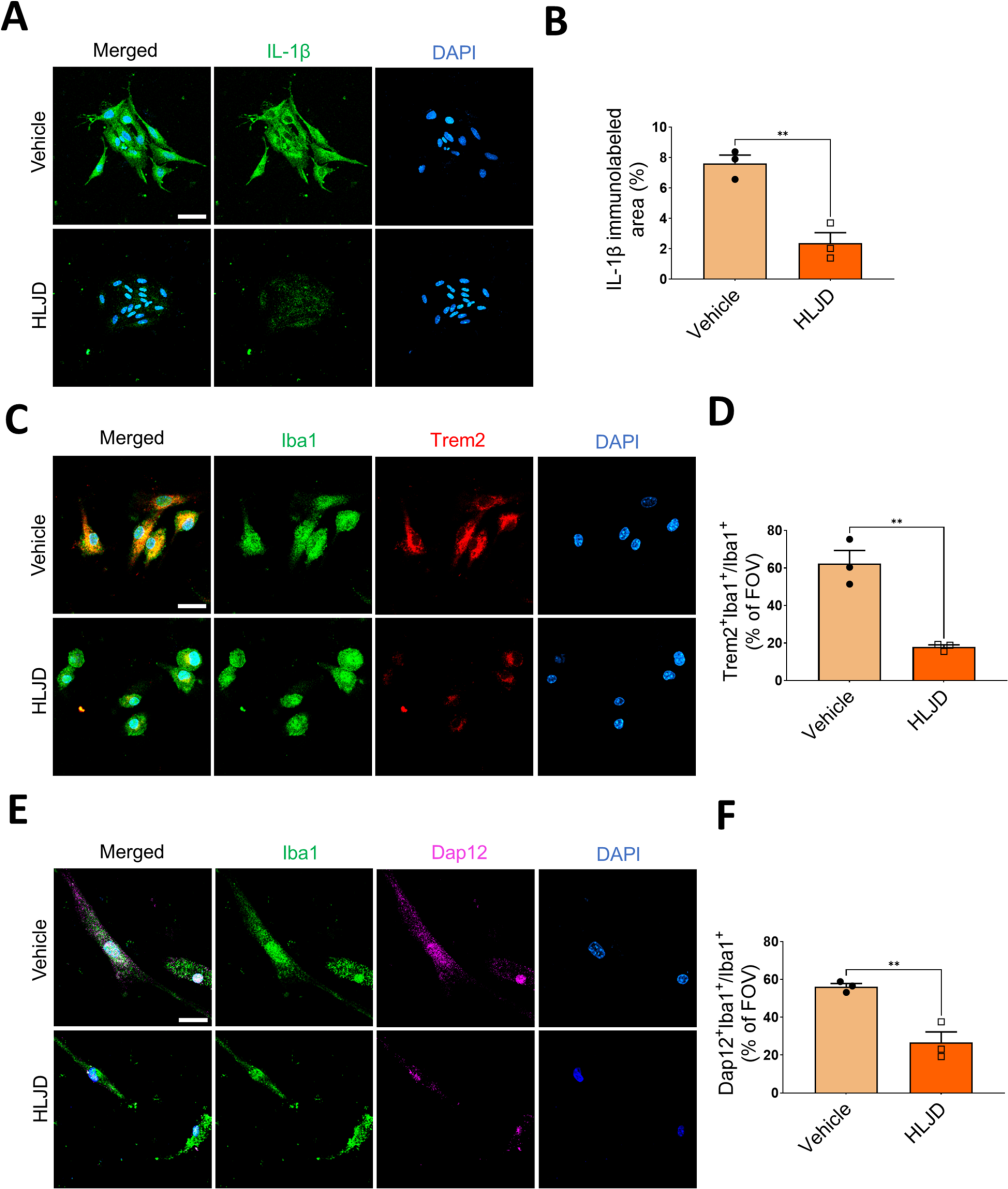
HLJD decoction inhibited the Trem2/Dap12 signaling pathway in the primary microglia of 5xFAD mice. **A** Representative images of IL-1β in the primary microglia of 5xFAD mice. Scale bars, 25 µm. **B** Quantification of IL-1β immunolabeled area. **C** Representative images of Trem2 and Iba1 staining in the primary microglia of 5xFAD mice. Scale bars, 25 µm. **D** Percentage of Trem2^+^Iba1^+^ cells in Iba1^+^ cells. **E** Representative images of Dap12 and Iba1 staining in the primary microglia of 5xFAD mice. Scale bars, 25 µm. **F** Percentage of Dap12^+^Iba1^+^ cells in Iba1^+^ cells (n = 3). Data are presented as mean ± SEM. **P < 0.01

To further confirm the effects of HLJD on inflammation conditions and the Trem2/Dap12 signaling pathway in microglia, we extracted primary microglia from the brains of 5xFAD mice and treated these cells with HLJD or vehicle in vitro. IF staining revealed that the expression of IL-1β in primary microglia treated with HLJD was significantly suppressed compared to that in vehicle-treated primary microglia (*P* < 0.01) (Fig. 9A, B), suggesting that HLJD decoction reduced the inflammatory cytokine levels in microglia. Additionally, the expression levels of Trem2 and Dap12 were significantly inhibited in HLJD-treated primary microglia from 5xFAD mice compared to vehicle-treated cells (*P* < 0.01) (Fig. 9C–F). These results indicate that HLJD decoction inhibited the Trem2/Dap12 signaling pathway, and alleviating microglial inflammation in vitro.

## Discussion

AD is the leading cause of dementia among middle-aged and elderly individuals, with its pathogenesis involving genetic and sporadic pathological mechanisms [36, 37]. There is an urgent need to identify effective therapeutic agents for AD treatment. Traditional Chinese medicine (TCM) has gained prominence as a potential alternative therapy in treating AD. Our data demonstrated that HLJD decoction effectively alleviated cognitive deficits in AD mouse model 5xFAD mice, which may be associated with its inhibition on Trem2/Dap12-associated microglial neuroinflammation.

5xFAD mice are commonly used animal model for AD study, that was established by overexpression of APP and PSEN1 gene containing five familial AD-related mutations [38]. 5xFAD mice exhibit progressive deposition of amyloid plaques, neuroinflammation, glial proliferation, neuronal loss accompanied by cognitive impairments [39, 40]. Extracellular Aβ plaques and intracellular neurofibrillary tangles containing hyperphosphorylated tau protein represent the two primary pathological features of AD [41]. Although anti-Aβ agents in clinical trials can effectively reduce amyloid plaques levels, their clinical benefits remain unclear [37]. This uncertainty underscores the complexity of AD etiology and the challenges associated with treatment options. In this study, HLJD was administered to 5xFAD mice at doses of 2 g/kg and 4 g/kg, which were selected based on previous research where HLJD doses ranged from 2 g/kg to 9.1 g/kg [20, 42–46]. Specifically, HLJD decoction at doses of 2, 4, and 8 g/kg has been shown to improve learning and memory following transient cerebral ischemia in mice [46]. Our data revealed that HLJD at both low and high doses improved cognitive impairments in 5xFAD mice, as evidenced by the MWM results (Fig. 1). Both doses of HLJD showed comparable efficacy in reducing Aβ and tau levels (Fig. 2).

Neuroinflammation constitutes a significant pathological factor in the progression of AD [47]. The activation of glial cells, release of pro-inflammatory factors, and neuronal damage create a vicious cycle that exacerbates the effects of neuroinflammation in AD [48]. Recent studies have been shown that neuroinflammation is not only a consequence of neurodegeneration but also a contributing factor in the progression of AD [49]. In fact, inflammation often precedes the deposition of amyloid plaques in neurodegenerative diseases [49, 50]. Neuroinflammation induced by genetic variations in the CNS or peripheral immune cells may trigger amyloid plaque deposition in certain susceptible populations, conversely, the deposition of amyloid plaques can subsequently stimulate the occurrence of inflammation [49, 51]. Activated microglia secrete inflammatory factors that disrupt the balance of M1 and M2 polarization, meanwhile they undergo morphological changes, transitioning from a ramified state to an activated “ameboid” shape [52]. Our study confirmed the observation of microglia activation, displaying as an expanding clustered distribution pattern in the HIP of 5xFAD mice. Previous research has demonstrated that neuroinflammation occurs in the HIP of 5xFAD mice, characterized by the excessive levels of TNF-α and IL-1β [53]. These findings are consistent with our data, showing that HLJD inhibited the elevation of TNF-α, IL-1β and IL-6 levels. These results suggested that HLJD may suppress inflammation in the HIP of 5xFAD mice, potentially contributing to the amelioration of cognitive deficits.

Network pharmacology analysis identified the Trem2/Dap12 signaling pathway as a key factor responsible for the effectiveness of HLJD in the treatment of AD. Trem2 is crucial for maintaining microglial homeostasis [54], through its association with Dap12, which is encoded by TYROBP gene [14]. Aβ plaques have been shown to bind to Trem2, thereby regulating microglia activation in neuroinflammation associated with AD [9]. ApoE, expressed in more than half of AD patients and working as the most prevalent genetic risk factor of AD, is also the ligand of Trem2 [55, 56]. APOE4 exacerbates Aβ aggregation, tau pathology, neuroinflammation, and AD progression [57]. DAM is a characteristic pathological feature in 5xFAD mice, exhibiting enhanced phagocytic function and a clustered distribution around Aβ plaques [17]. Previous studies have demonstrated that the activation of DAM is dependent on Trem2 in microglia [32, 58]. In the early stages of AD, DAM play a crucial role in the clearance of Aβ, however, in the late stages of the disease, the accumulation of amyloid plaques activates inflammasomes within microglia, leading to dysfunction of DAM and their involvement in the progression of the AD [17]. This phenomenon may be associated with the controversial roles of Trem2 in regulations of microglia function in AD. In the early stages, Trem2-positive microglia may facilitate the clearance of toxic proteins, whereas, in the late stage, Trem2 may contribute to neuroinflammation-related neuronal damage [59]. A study involving Trem2-deficient APP/PS1 mice supports this conclusion, showing that Trem2 deficiency exerts a disease stage-dependent effect on Aβ deposition and inflammatory conditions in the brain [60]. Moreover, a study using high-throughput flow cytometry identified a population of senescent microglia expressing high levels of Trem2 in a 7-month-old 5xFAD mouse model of amyloidosis, while Trem2-null mice exhibited a reduced number of senescent microglia [61]. In our study, HLJD was found to inhibit the Trem2/Dap12 signaling pathway, with the higher dose of HLJD demonstrating a more pronounced suppression of Trem2, suggesting a dose-dependent effect on this specific pathway.

When activated by Aβ plaques, tau protein, or other signals, microglia release various cytokines and chemokines that recruit monocytes from the bloodstream into the inflammatory tissue, which may play a significant role in the pathogenesis of AD [6]. Csf1 is an important cytokine that promotes the proliferation and differentiation of monocytes and microglia by binding to its receptor, Csf1r [33]. Trem2 may interact with the Csf1/Csf1r/Src signaling pathway, as Src is the key kinase responsible for phosphorylating the ITAM tyrosine residues on Dap12 when the ligand binds to Trem2 [62–64]. Furthermore, the increased presence of CD169 + macrophages in human and mouse gliomas indicates higher monocyte infiltration, resulting in the production of pro-inflammatory cytokines and chemokines [65]. In our study, we observed an increase in Csf1 and its receptor Csf1r, as well as the CD169 positive microglia in the HIP of 5xFAD mice. Treatment with HLJD decoction reduced the expression of Csf1, Csf1r, and CD169, suggesting that the increased infiltration of monocytes into the HIP of 5xFAD mice can be suppressed by HLJD decoction. In future studies, we aim to evaluate the therapeutic efficacy and the underlying mechanisms of the active components of HLJD in AD treatment. Additionally, we plan to investigate their potential synergistic interactions with clinically approved anti-AD therapeutics, such as donepezil or galantamine, through comprehensive in vitro and in vivo experiments. These studies may provide further insights into their therapeutic potentials for AD treatment.

## Conclusions

In summary, our data indicated that 10-month-old 5xFAD mice exhibit significant cognitive deficits, accompanied by the accumulation of Aβ, tau deposits, and microglial activation in the HIP. HLJD decoction can effectively alleviate cognitive impairment by inhibiting the Trem2/Dap12 signaling pathway related microglial neuroinflammation. This study suggested that HLJD decoction may work as a promising option for the treatment of cognitive deficits associated with AD.

## Supplementary Information

Supplementary Material 1.

Supplementary Material 2.

## Data Availability

Data will be made available on request.

## Abbreviations

AD: Alzheimer’s disease
Aβ: β-Amyloid
Csf1r: Colony-stimulating factor 1 receptor
CNS: Central nervous system
Dap12/TYROBP: DNAX-activating protein 12
DAM: Disease-associated microglia
ECL: Electrochemiluminescence
ELISA: Enzyme-linked immunosorbent assay
FBS: Fetal bovine serum
GSK3β: Glycogen synthase kinase-3 beta
GO: Gene Ontology
HIP: Hippocampus
HLJD: Huang-Lian-Jie-Du
Iba1: Ionized calcium-binding adaptor molecule 1
Il-1β: Interleukin-1β
Il-6: Interleukin-6
ITAM: Immunoreceptor tyrosine activation motif
MWM: Morris water maze
PPI: Protein–protein interaction
PVDF: Polyvinylidene fluoride
qPCR: Quantitative real-time PCR
RT: Room temperature
SDS-PAGE: Sodium dodecyl sulfate–polyacrylamide gel electrophoresis
Tau: Tubulin associated unit
TNF-α: Tumor necrosis factor-alpha
Trem2: Triggering receptor expressed on myeloid cells 2
TCM: Traditional Chinese medicine
TCMSP: Traditional Chinese Medicine Systems Pharmacology
ThS: Thioflavin S
WT: Wild type
SYK: Spleen tyrosine kinase
